# Temperature Responsive Nanoparticles Based on PEGylated Polyaspartamide Derivatives for Drug Delivery

**DOI:** 10.3390/polym11020316

**Published:** 2019-02-13

**Authors:** Guangyan Zhang, Xulin Jiang

**Affiliations:** 1 Hubei Provincial Key Laboratory of Green Materials for Light Industry, Hubei University of Technology, Wuhan 430068, China; 2 Key Laboratory of Biomedical Polymers of Ministry of Education & Department of Chemistry, Wuhan University, Wuhan 430072, China; xljiang@whu.edu.cn

**Keywords:** temperature responsiveness, polyaspartamide derivatives, click chemistry, drug delivery, polymeric nanoparticles

## Abstract

The temperature responsive PEGylated polyaspartamide derivative, denoted as mPEG-PAAHP, was synthesized by the click reaction. FTIR and ^1^H NMR were adopted to characterize and confirm the chemical structures of the obtained mPEG-PAAHPs. The temperature responsive behavior investigated by transmittance and dynamic light scattering showed that some of the obtained mPEG-PAAHPs exhibited obvious temperature responsiveness and could be used to prepare nanoparticles by quickly heating. Drug paclitaxel can be encapsulated into mPEG-PAAHP based nanoparticles with a high encapsulation efficiency up to 99% (corresponding to a drug loading content of around 9.9%). Dynamic light scattering results showed that the PTX-loaded nanoparticles had a mean size around 80 nm (PDI<0.2) and good stability in PBS with 150 mM ionic strength. In vitro cytotoxicity results showed that mPEG-PAAHP did not show any toxicity to HeLa cells, but the PTX-loaded nanoparticles based on mPEG-PAAHP exhibited obvious anti-cancer activity. Thus, the temperature responsive PEGylated polyaspartamide derivative mPEG-PAAHP may be a promising drug delivery system.

## 1. Introduction

Polymeric drug carriers with various stimuli responsiveness have attracted great attention in the past two decades [1,2]. These stimuli-responsive drug carriers usually can self-adjust their aggregation states or change their chemical structures when the external environment is changed [3,4], so the external environmental conditions, such as temperature [5,6] and pH [7,8,9], can be chosen as a key to implement drug loading or controlled drug release conveniently. For example, the hydrophobic drug paclitaxel (PTX) has been successfully encapsulated to prepare PTX-loaded nanoparticles just by heating the aqueous solution of temperature responsive polymers [10]. The entire drug-loading procedure occurs within five minutes and avoids the use of toxic organic solvents, and therefore shows a prominent advantage over the dialysis method. Thus, temperature responsive polymers exhibit great potential and prospects in drug delivery [11,12].

Compared with poly(N-isopropylacrylamide) and its based temperature responsive polymers [13,14], temperature responsive polymers derived from α-amino acids are considered more attractive [15], because the backbones of poly (amino acid)-based derivatives are biodegradable and their degradation products are expected to be non-toxic [16]. Up to now, three polyaspartamide anti-tumor formulations derived from *L*-aspartic acid (NK105 [17], NK911 [18] and NC6300 [19,20]) have been approved for clinical trials. The ring-opening reaction between poly(succinimide) (PSI) and amine compounds is an convenient strategy to synthesize polyaspartamide based polymers [21,22], and the stimuli responsiveness can be introduced by further modification of their side chains [23,24,25]. For example, the temperature responsive polyaspartamide based polymer phe-g-PHPA was prepared successfully by introducing phenyl alcohol (phe) to the side chains of PHPA, which was synthesized by the ring-opening reaction between PSI and 5-hydroxypentylamine, although the grafting efficiency of phe to PHPA was less than 50% [26,27].

Click chemistry usually possesses high reaction selectivity and efficiency, and has been widely applied in the biomedical field for preparing stimuli responsive polymers for anti-cancer drug delivery [2,28]. Based on the work related to PHPA, we designed an azide-functional polyaspartamide derivative P(Asp-Az)-HPA which was composed of about 40% 2-azidoethyl moiety and 60% 5-hydroxypentyl moiety in its side chains (molar percentage), and illustrated an easy way to synthesize temperature responsive polyaspartamide-based polymers by grafting hydrophobic moieties, such as phenyl alcohol, to the side chains of P(Asp-Az)-HPA via a click reaction with high grafting efficiency above 90% [29]. However, no real applications in drug delivery were mentioned for the obtained P(Asp-Az)-HPA-based temperature responsive polymers because of the low stabilities of their formed nanoparticles in salt conditions.

**Scheme 1:**
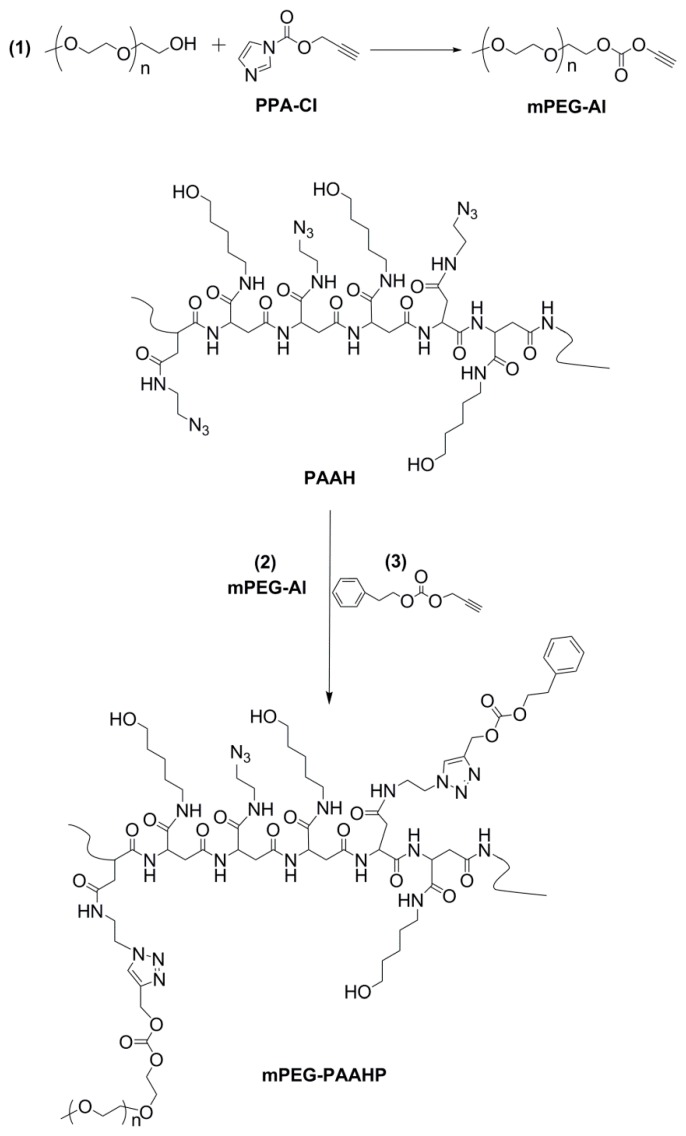
Synthesis route towards mPEG-PAAHP.

Hydrophilic long PEG chains can usually prevent non-specific interactions in blood and enhance the stability in salt conditions [30,31,32] or the mucus-penetrating properties [33] of nanoparticles. In this study, hydrophilic long PEG chains were introduced to P(Asp-Az)-HPA-based temperature responsive polymers via click chemistry. The designed PEGylated P(Asp-Az)-HPA-based temperature responsive polyaspartamide derivative, denoted as mPEG-PAAHP, was explored as depicted in Scheme 1. The preparation, characterization, drug loading, drug release, and in vitro cytotoxicity of mPEG-PAAHP based PTX-loaded polymeric nanoparticles were studied.

## 2. Materials and Methods

### 2.1. Materials

Phenyl alcohol, 1,1’-carbonyldiimidazole (CDI) and 4-dimethylaminopyridine (DMAP) were purchased from Shanghai Adamas Reagent Co., Ltd (Shanghai, China). 5-Hydroxypentylamine was purchased from Alfa Aesar. Propargyl alcohol (Wuhan FengFan Chemical Co., Ltd, Wuhan, China), *L*-Aspartic acid (Sinopharm Chemical Reagent Co., Ltd, Shanghai, China) and paclitaxel (Jiangsu Yew Pharmaceutical Co., Ltd, Wuxi, China) were used as received. Polyethylene glycol monomethyl ether (mPEG) was from Sigma-Aldrich (Louis, MO, USA) (*M*_n_ = 4431, *M*_w_/*M*_n_ = 1.28). Phenethyl prop-2-ynyl carbonate (PPA-PEA), 2-azidoethylamine and PSI were synthesized as reported in a previous study [29]. All other reagents and solvents were of analytical grade and used without further purification.

### 2.2. Synthesis of Mono-alkyne-functionalized Polyethylene Glycol monomethyl Ether

Propargyl ester of carbonylimidazole (PPA-CI) was synthesized by dropping propargyl alcohol into the suspension of CDI in dichloromethane. Mono-alkyne-functionalized polyethylene glycol monomethyl ether (mPEG-Al) was synthesized by the reaction between mPEG (2.0 g, 0.4 mmol) and excess propargyl ester of carbonylimidazole (3.0 g, 20 mmol) at 40 °C for 24 h using DMAP as catalyst and trichloromethane as solvent. The resulting reaction mixture was washed with water, and then the organic phase was dried with anhydrous MgSO_4_. The dried organic phase was dropped into ether and then filtered to obtain solid crude product. The crude product was further washed with ether three times and then vacuum-dried to obtain 1.61 g purified mPEG-Al. The structure of the obtained mPEG-Al was confirmed by ^1^H NMR as shown in Figure S1.

### 2.3. Synthesis of PEGylated Azide-functional Polyaspartamide Derivative

Azide-functional polyaspartamide derivative was synthesized according to our previous work [29]. The obtained product, P(Asp-Az)-HPA, abbreviated as PAAH in this paper, was composed of 39% 2-azidoethyl moiety and 61% 5-hydroxypentyl moiety in its side chains (molar percentage), calculated by its ^1^H NMR result in D_2_O.

PAAH (500 mg, about 50 equiv. polymer units) and mPEG-Al (257.7 mg, 1 equiv. alkyne groups) were dissolved in 15 mL DMF, then CuBr (1 equiv.) was added as catalyst under N_2_ atmosphere. The reaction mixture was stirred at 50 °C for 48 h. The resulting solution was dropped into ether slowly to collect the white precipitate, and then dried in vacuum to obtain the crude product. At last, the crude product was dissolved in 10 mL DMF and then further purified by dialysis against DI water for 4 days with a dialysis tube (MWCO 3.5 kDa), and the purified PEGylated azide-functional polyaspartamide derivative mPEG-PAAH was collected after lyophilization.

### 2.4. Synthesis of PEGylated Temperature Responsive Polyaspartamide Derivatives

PEGylated temperature responsive polyaspartamide derivative, denoted as mPEG-PAAHP, was synthesized by the click reaction between mPEG-PAAH (100 mg) and varying amounts of PPA-PEA (1 equiv.) in 10 mL DMF with CuBr (1 equiv.) as catalyst under N_2_ atmosphere. The reaction mixture was stirred at 50 °C for 48 h, and then dropped into ether slowly to collect the precipitate (crude product) and to remove unreacted PPA-PEA. At last, crude product dissolved in DMF was dialyzed against DI water under 4 °C for 1 week with a dialysis tube (MWCO 3.5 kDa). The purified PEGylated temperature responsive polyaspartamide derivatives mPEG-PAAHPs were collected after lyophilization.

### 2.5. Characterizations

^1^H nuclear magnetic resonance (^1^H NMR) spectra were recorded with a Mercury VX-300 spectrometer (300 MHz, Varian, Palo Alto, CA, USA). Fourier transformed infrared (FTIR) spectra were recorded on a Nicolet 6700 (Thermo Fisher, Hampton, NH, USA). The molecular weights and the molecular weight distributions of the obtained mPEG-PAAHPs were evaluated by size-exclusion chromatography-multi angle light scattering (SEC-MALLS, Milford, MA, USA) system consisted of a Waters 2690D separations module, a Waters 2414 refractive index detector (RI) and a Wyatt DAWN EOS MALLS detector. DMF containing 10 mM LiBr was used as the mobile phase at a flow rate of 0.3 mL/min at 30 °C. The data were processed with Astra software (Wyatt Technology, Santa Barbara, CA, USA).

Temperature responsive behaviors of mPEG-PAAHPs were evaluated by a transmittance measurement at 500 nm (Perkin-Elmer Lambda Bio 40 UV/Vis spectrometer, Hebron, KY, USA) and dynamic light scattering (DLS) (Nano ZS ZEN3600, Malvern Instruments, Westborough, MA, USA). The morphology of the nanoparticles was determined by DLS with a fixed scattering angle of 173° and by transmission electron microscopy (TEM). In this study, the cloud point (CP) was defined as the temperature corresponding to a 10% reduction in the initial transmittance of the solution.

### 2.6. Formation and Characterization of Nanoparticles

mPEG-PAAHP was dissolved in phosphate-buffered saline (PBS, pH 7.4, ionic strength 0.15 M) with a concentration of 2.0 mg/mL. Then, polymeric nanoparticles were prepared by quickly heating mPEG-PAAHP aqueous solution in a water bath at 60 °C. The size of the formed polymeric nanoparticles was determined by DLS.

The critical micelle concentration (CMC) of mPEG-PAAHP was determined by the fluorescent probe method with a fixed pyrene concentration of 6 × 10^−6^ M. The concentration of mPEG-PAAHP varied from 3.8 × 10^−6^ to 2.0 mg/mL. The excitation spectra were scanned from 300 to 360 nm with an emission wavelength of 390 nm. The intensity of I_337_ was analyzed as a function of the concentration of mPEG-PAAHP. The stability of the polymeric nanoparticles against PBS buffer dilution was investigated by DLS.

### 2.7. Drug Loading and Release

First, mPEG-PAAHP was dissolved in PBS (pH = 7.4, ionic strength 0.15 M) at a concentration of 2.0 mg/mL at 4 °C. Second, different volumes of PTX solution, dissolved in ethanol with a concentration of 10 mg/mL, were added into the mPEG-PAAHP solution. Next, the obtained mixtures were heated by soaking into a water bath immediately with 1 min incubation at 60 °C to form nanoparticles and to encapsulate PTX into the nanoparticles simultaneously. At last, the mixtures were slowly cooled down to room temperature. The non-entrapped or precipitated PTX was removed by filtration through a 220 nm filter. The amount of encapsulated PTX was determined by isocratic reversed-phase high-performance liquid chromatography (HPLC) (Labtech LC640 system, Beijing, China) using a reversed-phase Angel C18 column (250 × 4.6 mm^2^, 5 mm). The mixture of acetonitrile and water (60:40, *v*/*v*) was used as mobile phase with a flow rate of 0.6 mL/min, and UV detection was used for investigation at 227 nm. The calibration curve was also prepared for PTX dissolved in the mobile phase.

The drug release of PTX-loaded mPEG-PAAHP nanoparticles was evaluated under sink conditions by a dialysis method. PTX-loaded nanoparticles (PTX concentration, 0.1 mg/mL; mPEG-PAAHP concentration, 1.0 mg/mL) were prepared in PBS (pH = 7.4). Then, 1.2 mL of the PTX-loaded nanoparticles were placed into a dialysis tube (MWCO, 3.5 kDa) and immersed into 400 mL PBS containing 0.1% Tween 80 at 37 °C. Dialysis was performed at 37 °C for 24 h and the aqueous medium outside the dialysis tube was withdrawn periodically. The PTX concentration in different samples was also determined by HPLC as described above.

### 2.8. Cytotoxicity Assay

The cytotoxicity of the obtained mPEG-PAAHP was studied using the MTT assay against HeLa cells. The cells were seeded into 96-well plates at a density of 5000 cells/well and 100 μL Dulbecco’s modified Eagle’s medium (DMEM) containing 10% FBS was added. The cells were allowed to grow at 37 °C under a humidified atmosphere of 95% air and 5% CO_2_ for 24 h. Taxol was prepared as the comparison sample according to the method described by Soga et al. [10]. The cells were then incubated in culture media containing Taxol, Taxol vehicle (Taxol without PTX, Cremophor EL/ethanol=1/1, *v*/*v*), PTX-loaded nanoparticles and blank nanoparticles (without PTX), for a further 48 hours at 37 °C. Next, the medium was replaced with 200 μL fresh medium and the MTT reagent (20 μL in PBS, 5 mg/mL) was added to each well for a further 4 hours incubation at 37 °C. Then, the medium was removed and 100 μL of DMSO was added to dissolve the formed formazan crystals. The absorbance at 570 nm was recorded using Multiskan GO microplate spectrophotometer (Thermo Scientific, Hampton, NH, USA). The concentration of mPEG-PAAHP in blank nanoparticles was the same as that of PTX-loaded nanoparticles. After incubation, the relative cell viability (mean ± SD, *n* = 4) was calculated as: cell viability = (OD_sample_ − OD_blank_)/(OD_control_ − OD_blank_) × 100%, where OD_sample_ is the absorbance of solution with cells treated by the tested samples, OD_control_ is the absorbance for untreated cells (without mPEG-PAAHP), OD_blank_ is the absorbance without cells.

## 3. Results

In previous works, P(Asp-Az)-HPA was availed as a template to synthesize temperature responsive polymers by introducing hydrophobic moieties via the click reaction with a high reaction efficiency [29,34]. However, no real applications were mentioned for the obtained P(Asp-Az)-HPA-based temperature responsive polymers because of their low stabilities in the absence of hydrophilic long PEG chains. Here, PEGylated temperature responsive polyaspartamide derivatives containing pendant hydrophobic aromatic moieties (mPEG-PAAHPs) were synthesized as illustrated in Scheme 1 to investigate their properties for drug delivery. First, mono-alkyne-functionalized polyethylene glycol monomethyl ether mPEG-Al was synthesized. Then, mPEG was introduced to the side chains of azide-functional PAAH by the click reaction between PAAH and mPEG-Al to obtain the PEGylated azide-functional polyaspartamide derivative mPEG-PAAH. Lastly, phenethyl prop-2-ynyl carbonate was grafted to mPEG-PAAH via the click reaction to obtain temperature responsive polyaspartamide derivatives mPEG-PAAHPs.

### 3.1. Synthesis and Characterization of mPEG-PAAH

**Figure 1:**
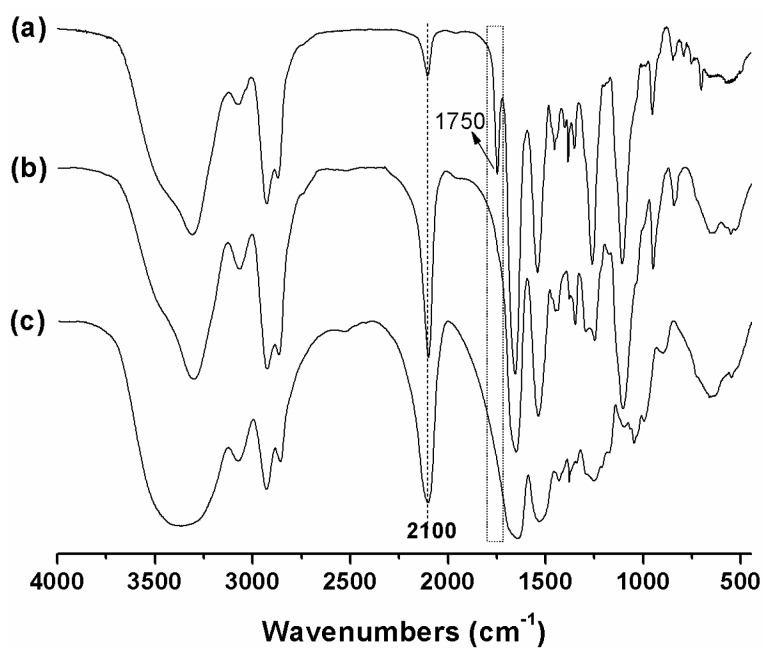
FTIR spectra of (**a**) mPEG-PAAHP-5, (**b**) mPEG-PAAH and (**c**) PAAH in KBr.

**Figure 2:**
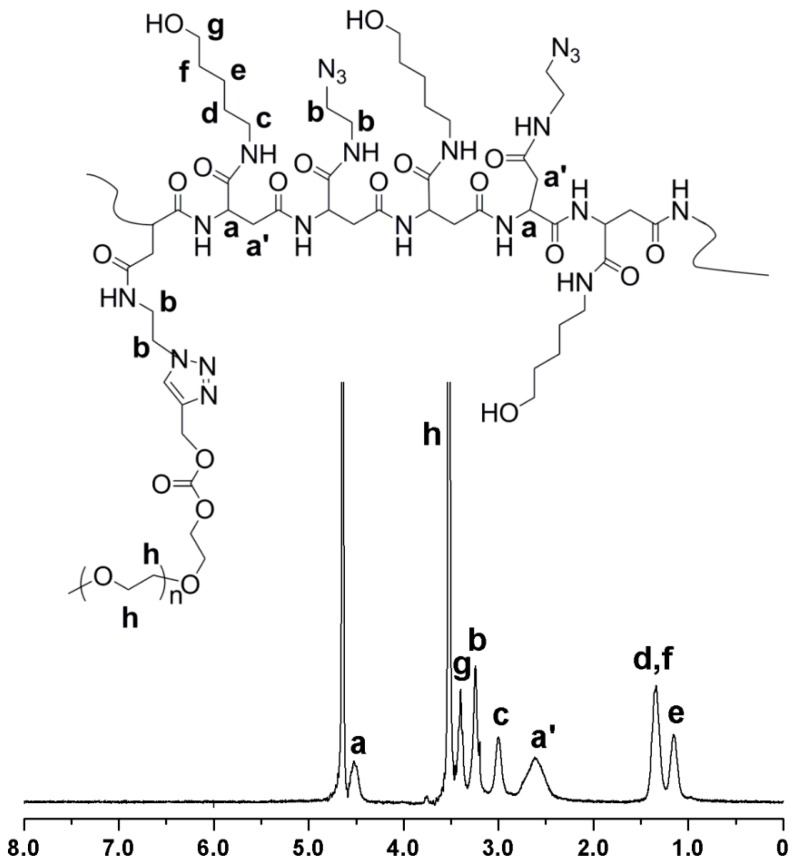
^1^H NMR spectrum of mPEG-PAAH in D_2_O.

FTIR results showed that the intensity of absorption peak at 2900 cm^−1^ corresponding to –CH_2_– in mPEG-PAAH (Figure 1b) was much higher than that of PAAH (Figure 1c), which implies that the content of methene groups in PAAH increases obviously after the click reaction between mPEG-Al and PAAH. These increased methene groups may be ascribed to the grafted mPEG. The ^1^H NMR spectrum of the PEGylated azide-functional polyaspartamide derivative mPEG-PAAH is shown in Figure 2, which also exhibits the characteristic peak of mPEG (3.5 ppm, h). The grafted ratio of mPEG is 2.0%, which was calculated based on the integral ratio of the proton peak h (3.5 ppm, methylenes in mPEG) and the proton peaks d, f and e (1.0–1.6 ppm, three methene groups in hydroxypentyl moiety). It is almost equal to the feed molar ratio of mPEG-Al/PAAH.

The weight average molecular weights and the molecular weight distributions of PSI, PAAH and mPEG-PAAH evaluated by SEC-MALLS using DMF as eluent, were 32.4 kDa (1.43), 47.4 kDa (1.72) and 53.6 kDa (1.64), respectively. The SEC traces of PSI, PAAH and mPEG-PAAH are shown in Figure S2, which indicates that the unreacted free mPEG-Al is negligible in the obtained mPEG-PAAH. It can be seen from the SEC-MALLS results that mPEG was grafted to the side chains of PAAH successfully, because the weight average molecular weight of mPEG-PAAH (53.6 kDa) was much higher than that of PAAH (47.4 kDa). With all FTIR, ^1^H NMR and SEC-MALLS analysis data taken together, it can be concluded that the conjugation of mPEG to the side chains of PAAH was successful and quantitative.

### 3.2. Synthesis and Characterization of Temperature Responsive mPEG-PAAHP

**Figure 3:**
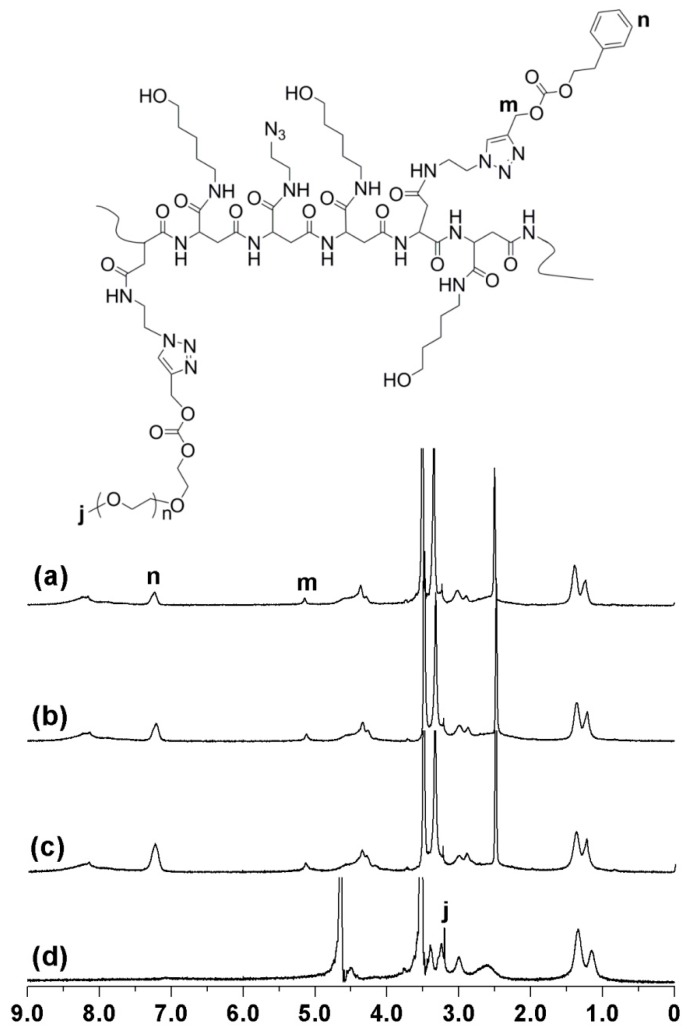
^1^H NMR spectra of (**a**) mPEG-PAAHP-2, (**b**) mPEG-PAAHP-3 and (**c**) mPEG-PAAHP-5 in DMSO-d_6_, and (**d**) mPEG-PAAHP-3 in D_2_O at 25 °C.

The structures of the obtained PEGylated temperature polyaspartamide derivatives mPEG-PAAHPs were characterized by FTIR (Figure 1a) and ^1^H NMR analysis (Figure 3). Compared to mPEG-PAAH, the absorption peak at 2100 cm^−1^ of mPEG-PAAHP became obviously weak (Figure 1a), which implies that the azide groups in the side chains of mPEG-PAAH reacted with the alkyne groups of PPA-PEA. Moreover, a new obvious absorption peak at 1750 cm^−1^ corresponding to the carbonyl groups in the PPA-PEA moieties of mPEG-PAAH was observed, which also implies that PPA-PEA successfully grafted to mPEG-PAAH.

The ^1^H NMR spectra of mPEG-PAAHPs are provided in Figure 3. It can be seen clearly from Figure 3 that two new peaks appear at 5.1 ppm (m) and 7.2 ppm (n, phenyl protons) (Figure 3a–c), which confirms the formation of triazole groups and the successful conjugation of hydrophobic PPA-PAE moieties, respectively. The molar percentage of grafted PPA-PEA in mPEG-PAAHP was calculated based on the ratio of the integral of 5.1 ppm (peak m, 2H) or 7.2 ppm (peak n, 5H of the benzene ring) and the integral from 1.0–1.6 ppm (6H). The results showed that the reaction efficiency between PPA-PEA and mPEG-PAAH was also high, above 90%, as our previous work reported [29] and summarized in Table 1. The molecular weights of mPEG-PAAHPs determined by SEC-MALLS were generally consistent with the ^1^H NMR results and are summarized in Table S1. Thus, the preparation of temperature responsive mPEG-PAAHP via the click reaction was successful.

The water solubility of the five obtained mPEG-PAAHPs containing varying amounts of hydrophobic PEA-PPA moieties showed that mPEG-PAAHP-5 was water insoluble even at 4 °C. The other four mPEG-PAAHPs [mPEG-PAAHP-1, mPEG-PAAHP-2, mPEG-PAAHP-3 and mPEG-PAAHP-4] were water soluble in a visual sense at 4 °C with an mPEG-PAAHP concentration of 2.0 mg/mL. However, no obvious temperature responsive behavior was observed for mPEG-PAAHP-1, because it was still water soluble even at 60 °C. Thus, the responsive behaviors of mPEG-PAAHP-2, mPEG-PAAHP-3 and mPEG-PAAHP-4 to temperature were investigated by DLS or transmittance.

**Figure 4:**
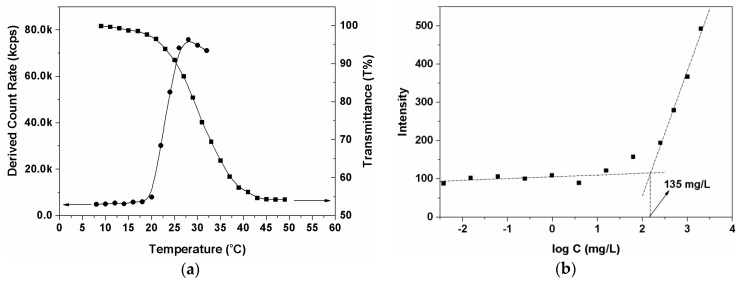
(**a**) The temperature responsive behavior of mPEG-PAAHP-3 aqueous solution in PBS (pH = 7.4, 2.0 mg/mL) in the heating process measured by UV/Vis at 500 nm (square) and by DLS (circle); (**b**) The intensity of the excitation spectra at 337 nm after excitation of mPEG-PAAHP-3 solutions at 390 nm as a function of logarithm of mPEG-PAAHP-3 concentration.

DLS results showed that the transitional process of mPEG-PAAHP-2 was gentle with a wide temperature range of 34 °C to 76 °C (Figure S3) though mPEG-PAAHP-2 responded to temperature variation. The temperature responsive behavior of mPEG-PAAHP-3 investigated by DLS showed that the cloud point of mPEG-PAAHP-3 was around 25 °C, which was very close to the value measured by transmittance (Figure 4a). For PEG-PAAHP-4 aqueous solution (2.0 mg/mL), it was transparent at a visual wavelength of 500 nm under 4 °C, but the DLS showed that most of PEG-PAAHP-4 existed as micelles in PBS because the derived count rate was high above ten thousand.

The critical micelle concentrations of mPEG-PAAHP-2, mPEG-PAAHP-3 and mPEG-PAAHP-4 were determined using pyrene as a fluorescent probe and are summarized in Table 1. It can be seen from Table 1 that CMC decreases with the increase of introduced hydrophobic PPA-PEA moieties in mPEG-PAAHPs. The CMC of mPEG-PAAHP-2, mPEG-PAAHP-3 and mPEG-PAAHP-4 at room temperature were 280 mg/L, 135 mg/L (Figure 4b) and 84 mg/L, respectively. Based on the results of transmittance, DLS and CMC, mPEG-PAAHP-2 and mPEG-PAAHP-3 were selected as representative samples for the following investigation for drug delivery.

### 3.3. Formation and Characterization of Nanoparticles

**Figure 5:**
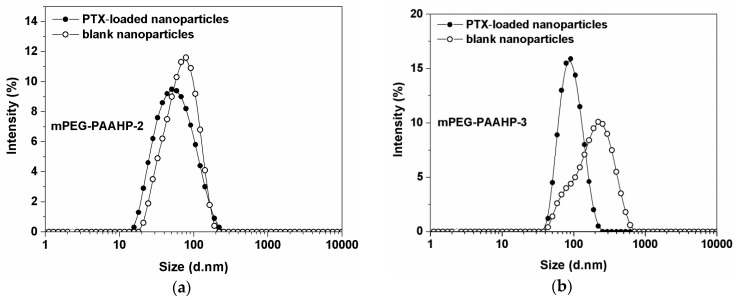
Diameters and size distributions of (**a**) mPEG-PAAHP-2 based blank nanoparticles and PTX-loaded nanoparticles (4.5%), and (**b**) mPEG-PAAHP-3 based blank nanoparticles and PTX-loaded nanoparticles (9.9%) in PBS buffer (pH = 7.4, ionic strength 0.15 M) at 37 °C. mPEG-PAAHP concentration = 2.0 mg/mL.

mPEG-PAAHP based nanoparticles were prepared by quickly heating mPEG-PAAHP aqueous solution to avoid the use of toxic organic solvents. The sizes and size distributions of mPEG-PAAHP-2 and mPEG-PAAHP-3 based nanoparticles, prepared by such a quick heating process from 4 °C to 60 °C, were determined by DLS at 37 °C. As shown in Figure 5, the sizes of mPEG-PAAHP-2 and mPEG-PAAHP-3 based blank nanoparticles in PBS buffer (pH = 7.4, ionic strength 0.15 M) were 65 nm and 160 nm, respectively.

The formation of mPEG-PAAHP based nanoparticles was further investigated and confirmed by ^1^H NMR (Figure 3d). As can be seen from Figure 3d, only the proton signals of hydrophilic moieties (mPEG and 5-hydroxypentyl moieties) were observed in D_2_O for mPEG-PAAHP-3, but no proton signals of hydrophobic moieties (PPA-PEA moieties) were observed. However, the phenyl protons signals were observed clearly at 7.2 ppm in DMSO-d_6_ for mPEG-PAAHP-3 as shown in Figure 3b. The absence of the phenyl proton signals indicated that the motion of the PPA-PEA moieties in the core was restricted, resulting in fast spin relaxation times that could not be detected using liquids as test condition [35,36]. Thus, the ^1^H NMR results of mPEG-PAAHP-3 in different solvents also confirmed the formation of nanoparticles, where hydrophobic PPA-PEA moieties were confined to the core, whereas other hydrophilic moieties (PEG and 5-hydroxypentyl moieties) were dissolved as shell in D_2_O.

### 3.4. Drug Loading and Release

Paclitaxel was encapsulated into mPEG-PAAHP nanoparticles by quickly heating the mixture of PTX solution in ethanol and mPEG-PAAHP solution in PBS for 1 minute in a water bath at 60 °C. The obtained PTX-loaded nanoparticles are visualized in Figure S4. The resulted homogeneous PTX-loaded nanoparticles dispersion was filtered through a 220 nm filter to remove possible non-entrapped PTX precipitates, and then analyzed by HPLC. HPLC analysis revealed that most of the PTX fed was loaded into mPEG-PAAHP based nanoparticles. The drug loading capacities of mPEG-PAAHPs are summarized in Table 2. As summarized in Table 2, the drug-loading content of mPEG-PAAHPs based nanoparticles can be adjusted from 4.5% to around 21% with increasing the PTX/mPEG-PAAHP feed weight ratio, but the PTX entrapment efficiency decreased with the increase of the PTX/mPEG-PAAHP feed weight ratio. However, mPEG-PAAHP-2 based PTX-loaded nanoparticles with 10% drug feeding were unstable and disintegrated within 30 min at 37 °C, which may be caused by the high cloud point and wide transition temperature range (34–76 °C) of mPEG-PAAHP-2. Thus, mPEG-PAAHP-2 based PTX-loaded nanoparticles with 5% drug feeding (LC: 4.5%) were used for drug release study. Additionally, mPEG-PAAHP-3 based PTX-loaded nanoparticles with 10% drug feeding were also used for the following drug release and cytotoxicity studies because the drug loading content of Taxol was 10%.

The average diameter and size distribution of mPEG-PAAHP based PTX-loaded nanoparticles were investigated by DLS in PBS. Compared with mPEG-PAAHP-3 based blank nanoparticles, the PTX-loaded nanoparticles (with LC 9.9%) exhibited a smaller diameter around 80 nm with a narrow size distribution (PDI < 0.2) obviously as shown in Figure 5b. A similar phenomenon was also observed on mPEG-PAAHP-2. These may be ascribed to the π-π stacking interaction between PTX and the hydrophobic phenyl moieties of mPEG-PAAHP, which enhanced the acting force in the core of nanoparticles and made nanoparticles compact and uniform. In addition, mPEG-PAAHP-3 based PTX-loaded nanoparticles exhibited good features in size and PDI under different salt concentrations in water (Figure S5) and excellent stability against PBS buffer dilution (Figure S6). The morphology investigated by TEM also confirmed the formation of mPEG-PAAHP-3 based nanoparticles as shown in Figure S7.

**Figure 6:**
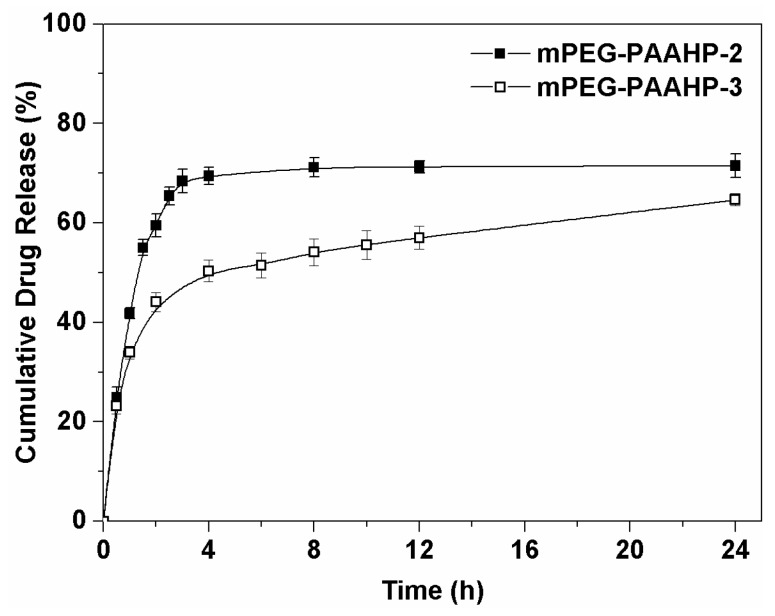
In vitro release profiles of mPEG-PAAHP-2 based nanoparticles containing 4.5% PTX (closed square) and mPEG-PAAHP-3 based nanoparticles containing 9.9% PTX (open square) at 37 °C in PBS (pH = 7.4). Error bars are standard deviations of three measurements.

The release behavior of PTX from PTX-loaded mPEG-PAAHP based nanoparticles was evaluated by a dialysis method in PBS (pH = 7.4) with 0.1% Tween 80 at 37 °C (Figure 6). Although the drug-loading content of mPEG-PAAHP-3 based PTX-loaded nanoparticles (9.9%) is more than two times that of mPEG-PAAHP-2 based PTX-loaded nanoparticles (4.5%), the percentage of released PTX from mPEG-PAAHP-3 based nanoparticles is obviously lower than that of mPEG-PAAHP-2 based nanoparticles.

After 24 h, HPLC analysis indicated that the remaining PTX of mPEG-PAAHP-2 (LC: 4.5%) and mPEG-PAAHP-3 (LC: 9.9%) based nanoparticles in the dialysis tube were 0.8% and 5.2% of the initial amount of loaded PTX, respectively. Thus, in drug release experiments, only about 70% of the initial loaded PTX was detected in total from both mPEG-PAAHP-2 and mPEG-PAAHP-3 based nanoparticles by HPLC. Soga et al. reported that there was 75% of initial PTX detected by HPLC after 20 hours drug release [10], and Wang et al. also reported that only about 60% of initial PTX was determined by HPLC [37]. They suggested that this incomplete PTX recovery may be caused by the adsorption of PTX onto the dialysis bag or the glass wall. Therefore, this also might be an explanation for the immense difference between the loaded PTX in the drug loading process and the PTX measured in the drug release process.

Considering the results of drug loading, DLS and drug release comprehensively, increasing the content of hydrophobic phenyl moieties in mPEG-PAAHP may be advantageous to enhance the π-π stacking interaction between PTX and mPEG-PAAHP, which might improve the drug loading capability of mPEG-PAAHP, stability of mPEG-PAAHP based nanoparticles and the sustained release of drug from mPEG-PAAHP based nanoparticles.

### 3.5. Cytotoxicity of mPEG-PAAHP based Nanoparticles

**Figure 7:**
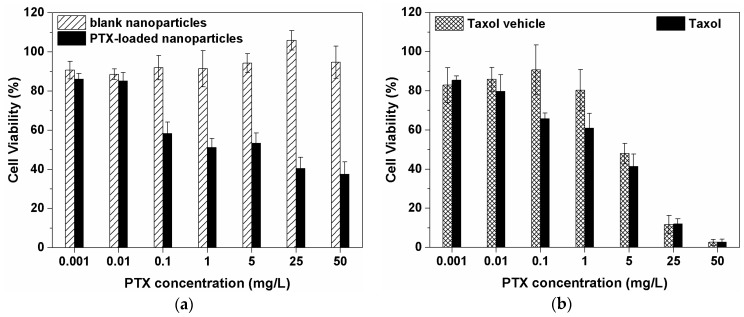
In vitro cytotoxicity of (**a**) mPEG-PAAHP-3 based PTX-loaded nanoparticles and blank nanoparticles, and (**b**) Taxol and Taxol vehicle on HeLa cells after 48 h of incubation. Data represent the mean and standard deviation of four independent experiments.

The MTT assay was carried out to evaluate the cytotoxicity of mPEG-PAAHP-3 based PTX-loaded nanoparticles (LC: 9.9%) and blank mPEG-PAAHP-3 based nanoparticles against HeLa cells (Figure 7a). In addition, the cytotoxicity of Taxol (LC: 10%) and the Taxol vehicle were also evaluated for comparison as shown in Figure 7b. As can be seen from Figure 7a, mPEG-PAAHP-3 based PTX-loaded nanoparticles exhibit cytotoxic activity against HeLa cells and the IC_50_ of PTX in mPEG-PAAHP-3 based PTX-loaded nanoparticles is about 1 mg/L. It is worth noting that mPEG-PAAHP-3 based blank nanoparticles do not show any toxicity even at the highest concentration of around 500 mg/L mPEG-PAAHP (the corresponding concentration of PTX: 50 mg/L). Although Taxol obviously exhibited a stronger cytotoxic activity against HeLa cells over mPEG-PAAHP-3 based PTX-loaded nanoparticles when the PTX concentration was above 5 mg/L, the Taxol vehicle exhibited a similar cytotoxic activity to Taxol as shown in Figure 7b. Therefore, for Taxol, the cytotoxicity may be ascribed to the Taxol vehicle rather than PTX; for mPEG-PAAHP-3 based PTX-loaded nanoparticles, the cytotoxicity is mainly ascribed to the encapsulated PTX.

## 4. Conclusions

A series of temperature responsive PEGylated polyaspartamide derivatives containing pendant phenyl moieties [mPEG-PAAHPs] were synthesized via the click reaction successfully. The structures of obtained mPEG-PAAHPs were confirmed by FTIR and ^1^H NMR. Transmittance and dynamic light scattering results showed that mPEG-PAAHP-3 exhibited temperature responsiveness obviously around 25 °C. PTX-loaded nanoparticles based on mPEG-PAAHP-3 were prepared by the quick heating method with an average diameter around 80 nm and a narrow size distribution (<0.2) in PBS, and exhibited good stability against PBS buffer dilution with 150 mM ionic strength. The PTX-loaded nanoparticles based on mPEG-PAAHP-3 showed obvious anti-cancer activity against HeLa cells, while empty nanoparticles based on mPEG-PAAHP-3 were non-toxic even at the concentration of 500 mg/L. The present results suggest that temperature responsive PEGylated polyaspartamide derivative mPEG-PAAHP may be a promising delivery system for the hydrophobic anticancer drugs.

## Figures and Tables

**Table 1:** Characterizations of mPEG-PAAHPs.

Sample	Molar Feed Ratio of PPA-PEA to the Polymer Unit of mPEG-PAAH	Percentage of Grafted PPA-PEA in mPEG-PAAHP ^1^	CMC (mg/L)	CP ^2^ (2 mg/mL)
mPEG-PAAHP-1	5%	4.8%	soluble	-
mPEG-PAAHP-2	10%	9.7%	280	MBR
mPEG-PAAHP-3	15%	14.5%	135	25 °C
mPEG-PAAHP-4	20%	17.8%	84	-
mPEG-PAAHP-5	30%	29.5%	insoluble	-

^1^ Calculated from ^1^H NMR spectra in DMSO-d_6_, ^2^ Determined in water by DLS. MBR=mild and broad response to temperature.

**Table 2:** PTX loaded into mPEG-PAAHPs based nanoparticles with varying drug feeding

Sample	Drug Feeding (Drug/mPEG-PAAHP, w%)	LC (%) ^a^	EE (%) ^b^
mPEG-PAAHP-2	5	4.5	90
mPEG-PAAHP-2	10	-	-
mPEG-PAAHP-3	10	9.9	99
mPEG-PAAHP-3	15	13.8	92
mPEG-PAAHP-3	20	17.5	88
mPEG-PAAHP-3	25	20.8	83

^a^ Drug loading content (LC) = (amount of PTX in the filtered nanoparticles/amount of mPEG-PAAHP) × 100%. ^b^ Drug entrapment efficiency (EE) = (amount of PTX in the filtered nanoparticles/amount of PTX in feed) × 100%. Note: mPEG-PAAHP concentration = 2.0 mg/mL.

## Supplementary Materials

The following are available online at http://www.mdpi.com/2073-4360/11/2/316/s1, Figure S1: ^1^H NMR spectrum of mPEG-Al in CDCl_3_, Figure S2: The SEC traces of PSI, PAAH and mPEG-PAAH, Figure S3: The temperature responsive behavior of mPEG-PAAHP-2 aqueous solution in PBS (pH = 7.4, 2.0 mg/mL) in the heating process measured by DLS, Figure S4: 20 μL PTX-containing ethanol solution (10 mg/mL) was added into PBS without mPEG-PAAHP-3 (A), and with 2 mg/mL mPEG-PAAHP-3 (B) by quick heating method before filtering through a 220 nm filter, Figure S5: Stability of mPEG-PAAHP-3 nanoparticles containing 9.9% loaded PTX in water at various salt concentrations by DLS at 37 °C, Figure S6: The effect of the concentration of mPEG-PAAHP-3 on the size and polydispersity of mPEG-PAAHP-3 based nanoparticles containing 9.9% PTX in PBS at 37 °C, Figure S7: The morphology of PTX-loaded nanoparticles based on mPEG-PAAHP-3 in PBS by TEM, Table S1: Molecular weights of mPEG-PAAHPs.
