# Supplementary material for: Temperature Responsive Nanoparticles Based on PEGylated Polyaspartamide Derivatives for Drug Delivery
Source: Polymers (Basel). 2019 Feb 13;11(2):316. doi: 10.3390/polym11020316 (PMC6419189; doi:10.3390/polym11020316)
Supplement: Supplementary file 1 [file polymers-11-00316-s001.pdf]

Supplementary Materials

Temperature Responsive Nanoparticles based on PEGylated Polyaspartamide Derivatives for Drug Delivery

Guangyan Zhang [1,2,*] and Xulin Jiang [2]

[1] Hubei Provincial Key Laboratory of Green Materials for Light Industry, Hubei University of Technology, Wuhan 430068, P.R. China; zhangguangyan@whu.edu.cn (G.Z.)
[2] Key Laboratory of Biomedical Polymers of Ministry of Education & Department of Chemistry, Wuhan University, Wuhan 430072, P.R. China; xljiang@whu.edu.cn (X.J.)
* Correspondence: zhangguangyan@whu.edu.cn (G.Z.)

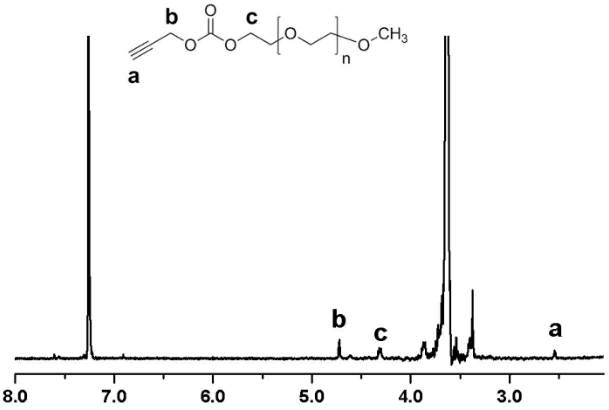

Figure S1. ^{1}H NMR spectrum of mPEG-Al in $CDCl_3$

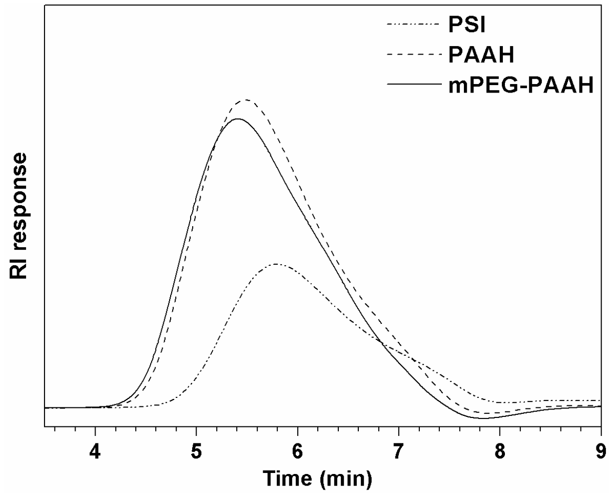

Figure S2. The SEC traces of PSI, PAAH and mPEG-PAAH.

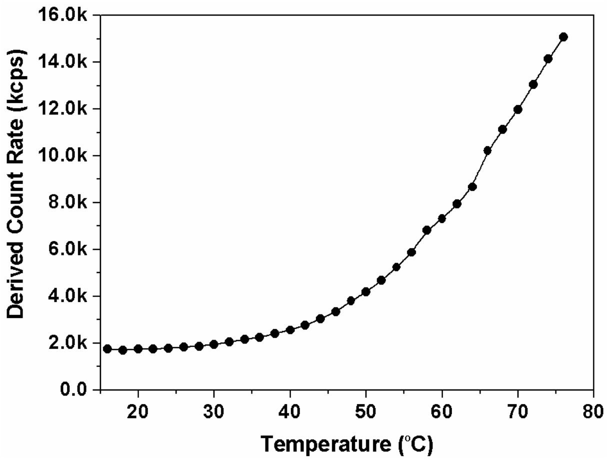

Figure S3. The temperature responsive behavior of mPEG-PAAHP-2 aqueous solution in PBS (pH=7.4, 2.0 mg/mL) in the heating process measured by DLS.

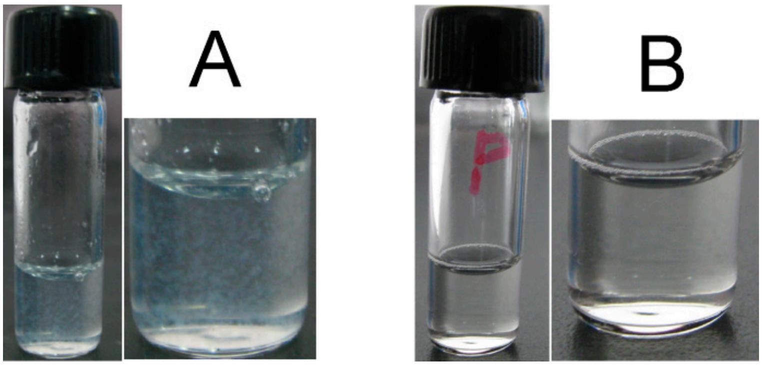

Figure S4. 20 μL PTX-containing ethanol solution (10 mg/mL) was added into PBS without mPEG-PAAHP-3 (A) and with 2 mg/mL mPEG-PAAHP-3 (B) by quick heating method before filtering through a 220 nm filter.

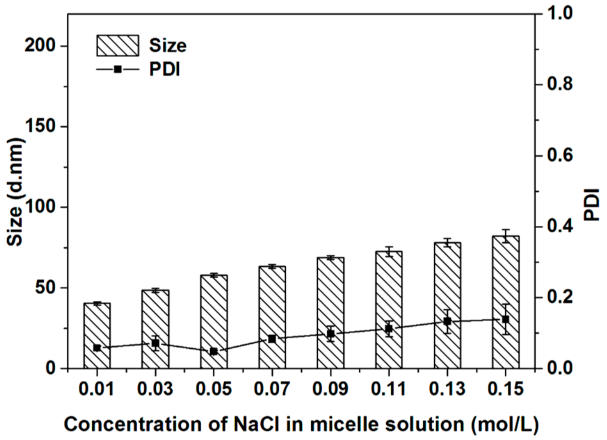

Figure S5. Stability of mPEG-PAAHP-3 nanoparticles containing 9.9% loaded PTX in water at various salt concentrations by DLS at 37°C. Data represent the mean and standard deviation of three independent experiments.

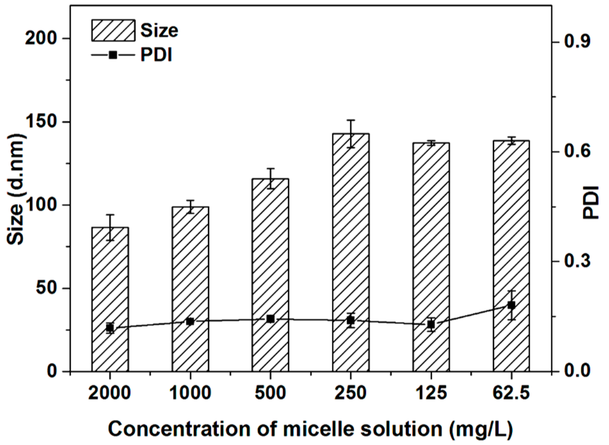

Figure S6. The effect of the concentration of mPEG-PAAHP-3 on the size and polydispersity of mPEG-PAAHP-3 based nanoparticles containing 9.9% PTX in PBS at 37°C. Data represent the mean and standard deviation of three independent experiments.

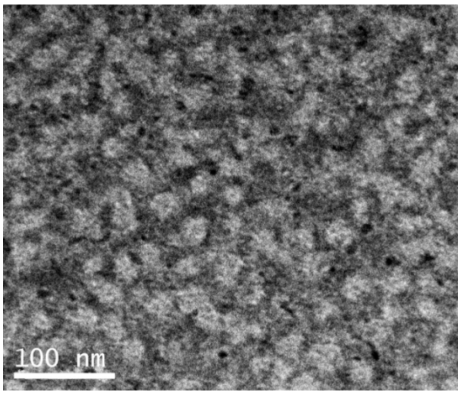

Figure S7. The morphology of PTX-loaded nanoparticles based on mPEG-PAAHP-3 in PBS by TEM.

Table S1. Molecular weights of mPEG-PAAHPs

Sample	M_n (×10³)	M_w (×10³)	M_w/M_n
mPEG-PAAHP-1	34	55	1.6
mPEG-PAAHP-2	35	57	1.6
mPEG-PAAHP-3	36	60	1.7
mPEG-PAAHP-4	39	64	1.6
mPEG-PAAHP-5	43	72	1.7
